# Computed tomography analysis of osteochondral defects of the talus after arthroscopic debridement and microfracture

**DOI:** 10.1007/s00167-015-3928-6

**Published:** 2015-12-28

**Authors:** M. L. Reilingh, C. J. A. van Bergen, L. Blankevoort, R. M. Gerards, I. C. M. van Eekeren, G. M. M. J. Kerkhoffs, C. N. van Dijk

**Affiliations:** Department of Orthopaedic Surgery, Orthopaedic Research Center Amsterdam, Academic Medical Center, University of Amsterdam, PO Box 22660, 1100 DD Amsterdam, The Netherlands

**Keywords:** Osteochondral defect, Subchondral bone, Cyst, Ankle, Arthroscopy, Microfracture

## Abstract

**Purpose:**

The primary surgical treatment of osteochondral defects (OCD) of the talus is arthroscopic debridement and microfracture. Healing of the subchondral bone is important because it affects cartilage repair and thus plays a role in pathogenesis of osteoarthritis. The purpose of this study was to evaluate the dimensional changes and bony healing of talar OCDs after arthroscopic debridement and microfracture.

**Methods:**

Fifty-eight patients with a talar OCD were treated with arthroscopic debridement and microfracture. Computed tomography (CT) scans were obtained at baseline, 2 weeks postoperatively, and 1 year postoperatively. Three-dimensional changes and bony healing were analysed on CT scans. Additionally, clinical outcome was measured with the American Orthopaedic Foot and Ankle Society (AOFAS) ankle-hindfoot score and numeric rating scales (NRS) for pain.

**Results:**

Average OCD size increased significantly (*p* < 0.001) in all directions from 8.6 (SD 3.6) × 6.3 (SD 2.6) × 4.8 (SD 2.3) mm (anterior–posterior × medial–lateral × depth) preoperatively to 11.3 (SD 3.4) × 7.9 (SD 2.8) × 5.8 (SD 2.3) mm 2 weeks postoperatively. At 1-year follow-up, average defect size was 8.3 (SD 4.2) × 5.7 (SD 3.0) × 3.6 (SD 2.4) mm. Only average defect depth decreased significantly (*p* < 0.001) from preoperative to 1 year postoperative. Fourteen of the 58 OCDs were well healed. No significant differences in the AOFAS and NRS-pain were found between the well and poorly healed OCDs.

**Conclusion:**

Arthroscopic debridement and microfracture of a talar OCD leads to an increased defect size on the direct postoperative CT scan but restores at 1-year follow-up. Only fourteen of the 58 OCDs were filled up completely, but no differences were found between the clinical outcomes and defect healing at 1-year follow-up.

**Level of evidence:**

IV.

## Introduction

An osteochondral defect (OCD) of the talus is a lesion of the cartilage and subchondral bone, mostly caused by a traumatic event. An OCD can either heal and become asymptomatic or progress to deep ankle pain [1–3].

Currently, arthroscopic debridement and microfracture is considered the primary treatment for symptomatic talar OCDs up to 15 mm in diameter [2, 4–7]. With this technique, all unstable cartilage including the underlying necrotic bone is removed. Following this, small holes are punctured in the subchondral bone to promote revascularization and induce bone and fibrous tissue formation [3]. Any underlying cyst(s) are opened, followed by curettage and perforation to release the pressure, which is assumed to stop further progression of the cyst [1, 3, 8].

Recently, there has been an increasing awareness of the role subchondral bone plays in the pathogenic process and development of deep ankle pain in OCDs. It is a prognostic factor for the eventual clinical outcome [2]. It has been suggested that an irregular subchondral bone plate may negatively affect articular cartilage repair in OCDs [9, 10]. Furthermore, structural changes in subchondral bone play a role in the pathogenesis of osteoarthritis [11, 12]. At long-term follow-up, progression of ankle osteoarthritis is seen in 33–34 % of patients following arthroscopic debridement and bone marrow stimulation of talar OCDs [13, 14]. Like in the knee, it was previously assumed that the bony defect would be filled primarily by bone, but this has never been investigated for the ankle [15–18]. The question of this study therefore is: to what extent the defect is filled with bone after debridement and microfracture? The hypotheses are [19] the defect dimensions will reduce 1 year after debridement and microfracture and [20] the defect dimensions shortly after the surgery are increased by debridement and microfracture. The secondary purpose is to determine whether defect size and bony healing are determinants of clinical outcome.

## Materials and methods

For this study, we used data from a randomized controlled trial investigating pulsed electromagnetic fields (PEMF) after arthroscopic debridement and microfracture of talar OCDs [21, 22].

Included were patients with a symptomatic talar OCD with a diameter smaller than 15 mm (in three dimensions), who were treated by arthroscopic debridement and microfracture at our centre. Both the PEMF treatment and placebo group were included, since no functional and radiological differences between the groups were found in the previous trial [21, 22]. Exclusion criteria were: age younger than 18 years, ankle osteoarthritis grade II or III [23], concomitant OCD of the tibia, ankle fracture less than 6 months before treatment of the OCD, surgical treatment of the index ankle performed <1 year before treatment of the OCD, concomitant painful or disabling disease of the lower limb, rheumatoid arthritis, and pregnancy.

### Operative technique

All arthroscopic procedures were performed using a standardized technique by one of the two experienced orthopaedic surgeons (GMMJK and CNvD). Anteromedial and anterolateral portals were created with the ankle in full dorsiflexion after which a 4-mm arthroscope was introduced. The OCD was identified with a probe by moving the ankle in full plantar flexion. After identification, all unstable bone and cartilage were removed with a curette and bonecutter shaver. Any underlying cyst(s) were opened, followed by curettage and perforation with a microfracture awl, with intervals of approximately 3 mm. At the end of the procedure, a pressure bandage was applied.

### Radiology

Computed tomography (CT) scans of the affected ankle were obtained preoperatively (mean 29 ± 7 weeks before surgery), at 2 weeks (mean 2 ± 1 weeks), and 1 year postoperatively (mean 53 ± 7 weeks). The scanning protocol involved “ultra-high-resolution” axial slices with an increment of 0.3 mm and a thickness of 0.6 mm, and multi-planar coronal and sagittal reconstructions of 1.0 mm [21, 22]. CT scanning has been proven to be accurate in the detection and follow-up of OCDs of the talus, regarding location and extent as well as healing of the defect [16–18].

The OCDs were graded on the preoperative CT scans according to the modified Berndt and Harty classification [20, 24].

Three-dimensional changes in the OCDs were evaluated by measuring the largest diameter (mm) in the anterior–posterior direction, medial–lateral direction and depth on each scan. The anterior–posterior size was measured at the level of the subchondral bone plate on the sagittal CT reconstruction. The depth was determined by drawing a circle through the subchondral bone plate of the talus on the sagittal CT reconstruction. A perpendicular line form the circle to the deepest point of the defect was measured as the depth. The medial–lateral size was measured on the coronal CT reconstruction from the most central point of the defect to the medial/lateral facet.

Different radiological aspects were assessed on the postoperative CT scans. The precision of addressing the entire OCD was evaluated on the two weeks postoperative CT scan. Cystic OCDs were specifically evaluated by assessing whether the cyst was opened and whether the cyst wall was perforated. The cyst was defined as opened when the wall of the cyst was disrupted on the two weeks postoperative CT scan in comparison with the preoperative CT scan. Formation of new cysts was evaluated on the one year postoperative CT scans. The presence of a new cyst was defined as a new radiolucent rounded area at final follow-up that was not visible on the two weeks postoperative CT scan. Healing of the subchondral bone was evaluated at final follow-up. Good healing was defined as complete osseous union or ossification, fair as incomplete osseous union or ossification but improvement compared with the preoperative findings, and poor as no changes between preoperative and postoperative [25]. All scans were analysed by a single physician who was blinded to the clinical outcomes. Measurements of the defect size were taken twice on the two weeks postoperative CT scans with an interval of 1 month and in different order to assess the intra-observer reliability.

### Clinical outcomes

Clinical outcomes were assessed with use of the American Orthopaedic Foot and Ankle Society (AOFAS) ankle-hindfoot score and numeric rating scales (NRS) for pain preoperatively and 1 year postoperatively [26, 27]. The AOFAS is a 100-point score, with a subjective and an objective component, which devotes 40 points to pain, 50 points to function, and 10 points to alignment [26, 27]. The NRS is comprised of an 11-point scale, which represents the spectrum of no pain (0 points) to the worst pain imaginable (10 points) [28].

The local Medical Ethics Committee at the University of Amsterdam approved the study with reference number MEC 08/326. The study is registered in the Netherlands Trial Register (NTR1636). Written informed consent was obtained from all participants.

### Statistical analysis

All analyses were performed in SPSS version 20.0 (Statistical Packages for Social Sciences Inc, Chicago, IL, USA). Categorical data are presented as frequencies. Continuous data are presented as mean with standard deviation (SD) or as median with interquartile range (IQR) depending on their distribution. Paired *t* test analyses were performed to determine the dimensional changes in the OCDs and clinical changes between the preoperative and postoperative CT scans. Pearson correlation test was used to analyse the correlation between the one year postoperative defect size and clinical outcome (AOFAS and NRS-pain). Independent *t* test was performed to analyse the differences in clinical outcome (AOFAS and NRS-pain) between a good and poorly healed subchondral bone and between defects with or without a cyst at final follow-up. A *p* value <0.05 was considered statistically significant. To assess the intra-observer reliability of the defect size measurements, the intra-class correlation coefficient (ICC) was calculated. An ICC of 0.85 or higher indicates good reliability [29, 30].

## Results

A total of 58 patients were included between 2009 and 2014 (Table 1). One patient did not undergo the final CT scan as she underwent a HemiCAP^®^ procedure during follow-up because of persisted deep ankle pain after a new ankle distortion [31].

**Table 1:** Baseline characteristics of the patients (*n* = 58)

Age (years), mean (SD)	32 (10)
Gender, *n* (% male)	35 (60)
BMI, mean (SD)	26 (4)
Smoking, *n* (%)	11 (19)
Ankle trauma, *n* (%)	41 (71)
Ankle fracture, *n* (%)	4 (7)
OCD operation of included side, *n* (%)	13 (22)
Side, *n* (% right)	32 (55)
Location, *n* (%)	
Medial	38 (66)
Lateral	17 (29)
Central	3 (5)
OCD classification, *n* (%)	
Compression	19 (33)
Completely undisplaced fracture	10 (17)
Displaced fracture	2 (3)
Cystic lesion	27 (47)

### Radiology

**Fig. 1:**
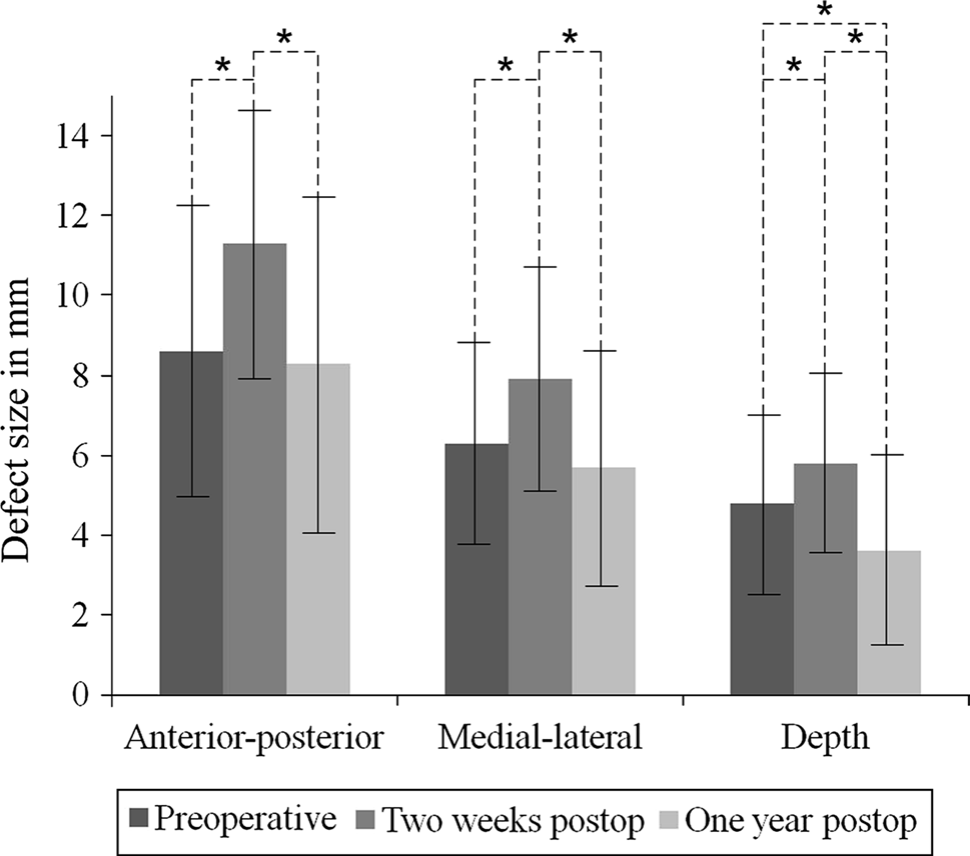
Mean defect size measured on the preoperative, two weeks postoperative, and one year postoperative CT scans. The *error bars* represent the standard deviation. Significant differences are indicated (*asterisk*)

**Fig. 2:**
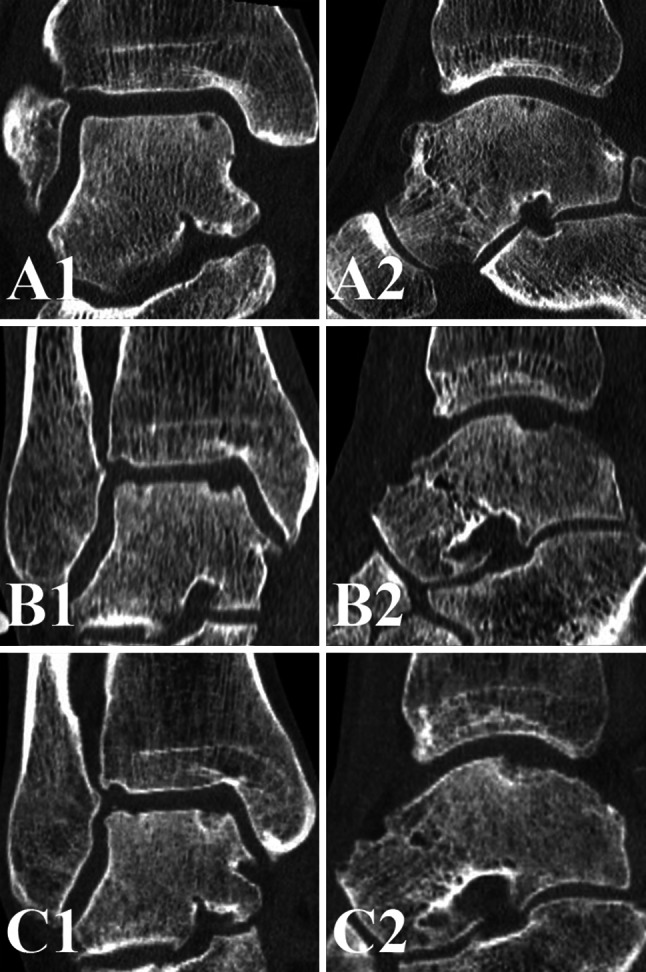
**a** Preoperative coronal (**A1**) and sagittal (**A2**) CT scans of a *right* ankle show a cystic OCD of the *medial* talar dome, **b** the two weeks postoperative coronal (**B1**) and sagittal (**B2**) CT scans show an increased defect size after technically successful debridement and microfracture, **c** at 1-year follow-up, the defect size decreased and the level of subchondral bone plate was almost flush [coronal CT scan (**C1**); sagittal CT scan (**C2**)]

Table 2 shows the defect size at baseline and follow-up. Two weeks after arthroscopic debridement and microfracture, the defect size increased significantly (*p* < 0.001) in all three dimensions. At 1-year follow-up, the defect size decreased significantly (*p* < 0.001) in all directions when compared to the two weeks postoperative defect size. No statistically significant differences in the anterior–posterior and medial–lateral directions were observed between the preoperative and one year postoperative CT scans. The depth decreased significantly (*p* < 0.001) from preoperative to 1 year postoperative (Figs. 1, 2). Intra-observer reliability of defect size measurements was good (ICC = 0.98).

**Table 2:** Three-dimensional defect size at baseline and after debridement and microfracture

	Preoperative	Two weeks postoperative	One year postoperative
Anterior–posterior, mean (SD)	8.6 (3.6)	11.3 (3.4)	8.3 (4.2)
Medial–lateral, mean (SD)	6.3 (2.6)	7.9 (2.8)	5.7 (3.0)
Depth, mean (SD)	4.8 (2.3)	5.8 (2.3)	3.6 (2.4)

**Fig. 3:**
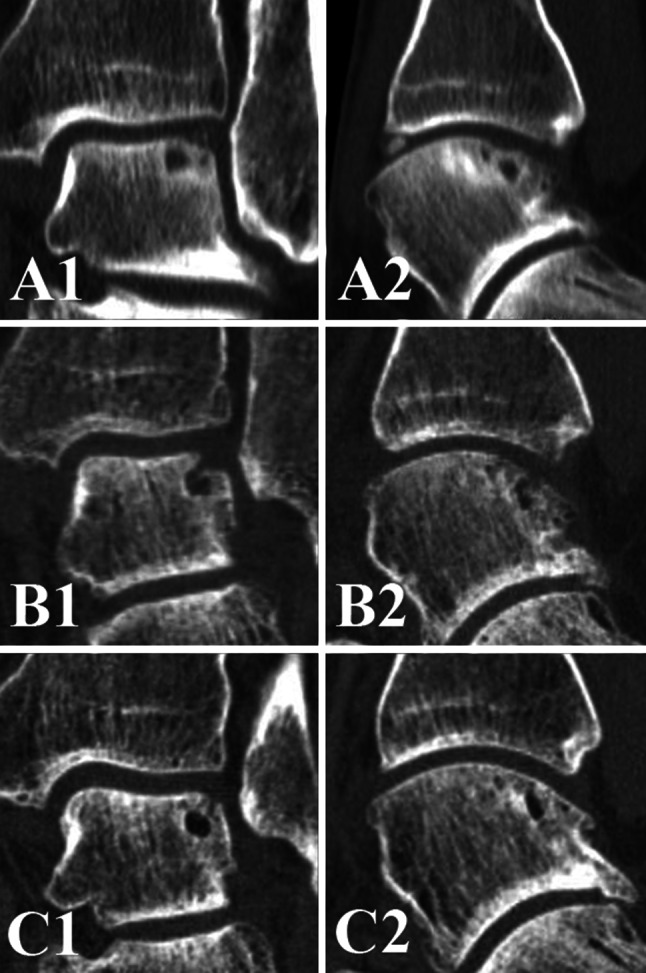
**a** Preoperative coronal (**A1**) and sagittal (**A2**) CT scans of a *left* ankle with a cystic OCD of the *lateral* talar dome, **b** the two weeks postoperative coronal (**B1**) and sagittal (**B2**) CT scans show that the cystic OCD is opened but not debrided, **c** at 1-year follow-up, the cyst is still visible [coronal CT scan (**C1**); sagittal CT scan (**C2**)]

**Fig. 4:**
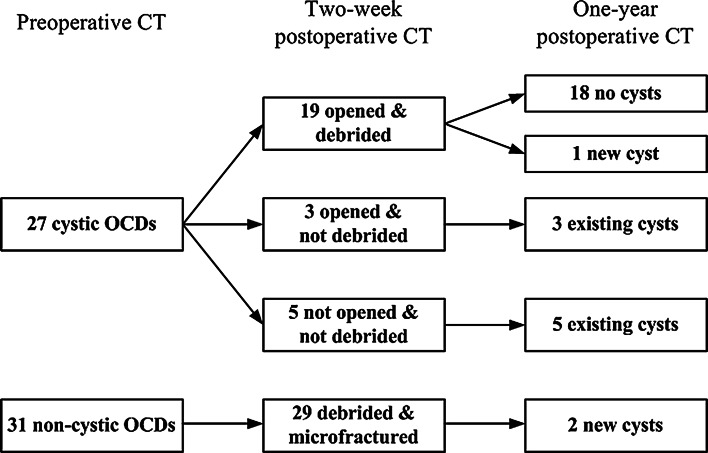
Follow-up of cyst formation after debridement and microfracture of OCDs of the talus. The two new cysts developed from a noncystic OCD were adequately treated during surgery

**Fig. 5:**
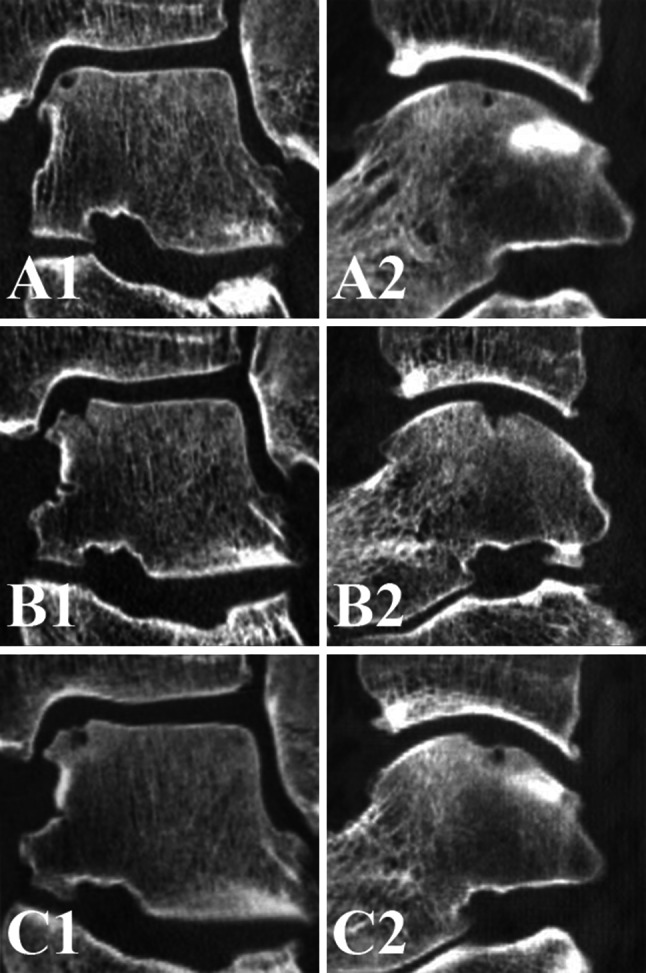
**a** Preoperative coronal (**A1**) and sagittal (**A2**) CT scans of a *left* ankle with a cystic OCD of the *medial* talar dome, **b** the two weeks postoperative coronal (**B1**) and sagittal (**B2**) CT scans show that the cystic OCD is opened and a microfracture hole is visible, **c** at 1-year follow-up, a new cyst has developed, possibly out of a microfracture hole [coronal CT scan (**C1**); sagittal CT scan (**C2**)]

Analysis of the precision of addressing the OCD revealed that 45 of 58 OCDs were treated adequately, two defects were not debrided, three defects were debrided partially, three cystic lesions were only opened at the roof, while the wall was not debrided (Fig. 3), and five cysts were not opened. The follow-up of postoperative cyst formation are presented in Fig. 4 and 5. The mean cyst size at final follow-up was 4.3 mm (SD 1.0) in the anterior–posterior direction, 4.1 mm (SD 0.7) in the medial–lateral direction, and 4.9 mm (SD 1.2) in depth.

Healing of the subchondral bone was good in 14 defects (25 %), fair in 22 defects (38 %), and poor in 21 defects (37 %).

### Clinical outcome

The preoperative mean AOFAS score improved from 58.8 (SD 14.2) to 84.5 (SD 17.3) at 1-year follow-up (*p* < 0.001). The mean NRS-pain in rest improved from 2.8 (SD 2.2) preoperatively to 1.1 (SD 1.8) at 1-year follow-up (*p* < 0.001). The mean NRS-pain when running improved from 8.0 (SD 1.9) preoperatively to 3.9 (SD 2.9) at 1-year follow-up (*p* < 0.001).

### Association between CT and clinical outcomes

The one year postoperative defect size did not correlate with the AOFAS and NRS-pain.

OCDs with a good subchondral bone healing at final follow-up had a mean AOFAS score of 85.6 (SD 17), a mean NRS-rest of 1.4 (SD 2.6), and a mean NRS-running of 4.5 (SD 3.6). OCDs with a poor healing at final follow-up had a mean AOFAS score of 84.0 (SD 19.4), a mean NRS-rest of 1.3 (SD 1.7), and a mean NRS-running of 3.7 (SD 3.1). No statistically significant differences in the AOFAS and NRS-pain were found between a good and poorly healed subchondral bone.

Defects with the presence of a cyst at final follow-up had a mean AOFAS score of 90.5 (SD 7.0), a mean NRS-rest of 1.3 (SD 1.7), and a mean NRS-running of 2.9 (SD 2.2). Defects without the presence of a cyst at final follow-up had a mean AOFAS score of 83.9 (SD 18.3), a mean NRS-rest of 1.1 (SD 1.9), and a mean NRS-running of 4.1 (SD 3.0). No statistically significant differences in the AOFAS and NRS-pain were found between defects with or without a cyst at final follow-up.

## Discussion

The most important findings of the present study are that 2 weeks after arthroscopic debridement and microfracture, the defect size increased significantly in all three dimensions. At 1-year follow-up, the defect size decreased significantly in comparison with the two weeks postoperative CT scans. In comparison with the preoperative defect size, only the depth of the defect decreased significantly at 1-year follow-up. Fourteen of the 58 OCDs were well healed. However, no differences were found between the clinical outcomes and defect healing or defect size.

In 1988, Zinman et al. [18] found 17 of 20 OCDs healed on CT scans after debridement and bone marrow stimulation or fixation of the fragment. Unfortunately, the definition of a healed OCD was not described in that study. Using plain radiographs, Kumai et al. [25] found a decreased defect size in 15 of 18 patients after debridement and microfracture at a mean follow-up of 4.6 years. However, healing of an OCD on plain radiographs is difficult to interpret because of overprojection of bone. Magnetic resonance imaging (MRI) is often used to quantify cartilage repair after debridement and microfracture [19, 32–34], but is less accurate to visualize bone. In this study, we focused on bone repair of an OCD, because the subchondral bone has an essential role in cartilage repair and in the pathogenesis of osteoarthritis [9–12].

In the present study, cystic OCDs were found in 27 out of 58 patients preoperatively. With the development of diagnostic methods MRI and CT, cystic lesions of the talus have been found to be present in 46–77 % of the chronic OCDs [13, 35]. When OCDs are treated nonoperatively, cyst development is seen in 12–14 % during follow-up [36, 37]. These new cysts have been correlated with an increased pain sensation [37]. The possible association between pain and new cyst formation might be caused by repetitive high fluid pressure during walking, which results in stimulation of the highly innervated subchondral bone underneath the cartilage defect [2, 3, 38]. In case the fissure through the subchondral bone plate is healed, it is assumed that no pressure builds up in the cyst, and the cyst will not grow [1, 3, 8]. In our study, 8 of the 11 cysts did not change in size between the two weeks and one year postoperative CT scans. This might explain why in our study, no significant differences in pain were found between defects with or without a cyst at final follow-up.

This is the first study that investigated the radiological changes after arthroscopic debridement and microfracture of talar OCDs at different time points. Strengths of this study include the complete radiological and clinical follow-up. Limitations include the two-dimensional analysis of a three-dimensional structure and the lack of long-term follow-up. A longer follow-up might show us the correlation between the degree of defect healing and progression of ankle osteoarthritis. Furthermore, MRI scans may give additional information on the quality of cartilage repair in comparison with CT scans.

Little is known about the bony healing of OCDs after arthroscopic debridement and microfracture. This study is useful as it suggests that no differences were found between the clinical outcomes and the degree of defect healing at 1-year follow-up.

## Conclusion

Arthroscopic debridement and microfracture of a talar OCD leads to an increased defect size on the direct postoperative CT scan but restores at 1-year follow-up. Only fourteen of the 58 OCDs were well healed, but no differences were found between the clinical outcomes and defect healing at 1-year follow-up.
